# EANM/EARL FDG-PET/CT accreditation - summary results from the first 200 accredited imaging systems

**DOI:** 10.1007/s00259-017-3853-7

**Published:** 2017-12-01

**Authors:** Andres Kaalep, Terez Sera, Wim Oyen, Bernd J. Krause, Arturo Chiti, Yan Liu, Ronald Boellaard

**Affiliations:** 1 Department of Medical Technology, North Estonia Medical Centre Foundation, J. Sutiste Str 19, 13419 Tallinn, Estonia; 2 Department of Nuclear Medicine, University of Szeged, Szeged, Hungary; 3 On behalf of EANM Research Limited (EARL), Vienna, Austria; 4 The Institute of Cancer Research, Division of Radiotherapy and Imaging, Department of Nuclear Medicine, The Royal Marsden Hospital, London, UK; 5 Department of Nuclear Medicine, Rostock University Medical Center, Rostock, Germany; 6 Department of Nuclear Medicine, Humanitas Clinical and Research Center, Rozzano, MI Italy; 7 European Organisation for Research and Treatment of Cancer (EORTC) Headquarters, Brussels, Belgium; 8 Department of Nuclear Medicine and Molecular Imaging, University of Groningen, University Medical Center Groningen, Hanzeplein 1, Groningen, the Netherlands; 9 Department of Radiology and Nuclear Medicine, VU University Medical Center, Amsterdam, The Netherlands

**Keywords:** Performance, Harmonisation, PET/CT, Quantification, EARL accreditation

## Abstract

**Purpose:**

From 2010 until July 2016, the EANM Research Ltd. (EARL) FDG-PET/CT accreditation program has collected over 2500 phantom datasets from approximately 200 systems and 150 imaging sites worldwide. The objective of this study is to report the findings and impact of the accreditation program on the participating PET/CT systems.

**Methods:**

To obtain and maintain EARL accredited status, sites were required to complete and submit two phantom scans - calibration quality control (CalQC), using a uniform cylindrical phantom and image quality control (IQQC), using a NEMA NU2–2007 body phantom. Average volumetric SUV bias and SUV recovery coefficients (RC) were calculated and the data evaluated on the basis of quality control (QC) type, approval status, PET/CT system manufacturer and submission order.

**Results:**

SUV bias in 5% (*n* = 96) of all CalQC submissions (*n* = 1816) exceeded 10%. After corrective actions following EARL feedback, sites achieved 100% compliance within EARL specifications. 30% (*n* = 1381) of SUVmean and 23% (*n* = 1095) of SUVmax sphere recoveries from IQQC submissions failed to meet EARL accreditation criteria while after accreditation, failure rate decreased to 12% (*n* = 360) and 9% (*n* = 254), respectively. Most systems demonstrated longitudinal SUV bias reproducibility within ±5%, while RC values remained stable and generally within ±10% for the four largest and ±20% for the two smallest spheres.

**Conclusions:**

Regardless of manufacturer or model, all investigated systems are able to comply with the EARL specifications. Within the EARL accreditation program, gross PET/CT calibration errors are successfully identified and longitudinal variability in PET/CT performances reduced. The program demonstrates that a harmonising accreditation procedure is feasible and achievable.

**Electronic supplementary material:**

The online version of this article (10.1007/s00259-017-3853-7) contains supplementary material, which is available to authorized users.

## Introduction

Positron emission tomography (PET) and computed tomography (CT) hybrid imaging (PET/CT) using ^18^F–fluorodeoxyglucose (FDG) has become a routinely used and valuable tool in oncology. It is widely utilised for diagnosis, staging and restaging of various malignancies [1–12] as well as response monitoring due to its ability to measure metabolic changes [13–19]. Standard uptake value (SUV), which represents the tissue radioactivity concentration normalised to injected activity and body weight [20] is the most frequently used quantitative metric in oncology [21, 22]. Multiple factors, however, can give rise to bias [23–25] and increased variability in SUV, especially when inter-centre comparison is required from institutions lacking a uniform approach to imaging procedures [26–28]. The variability is a significant issue for clinical trials or multicentre studies utilising the quantitative potential of PET [24, 26–31]. In clinical practice, there is a wide range of PET systems installed globally including scanners developed more than 10 years ago along with brand new devices incorporating state of the art acquisition (i.e., time of flight, digital PET detectors) and reconstruction (i.e., resolution modelling) technologies [32]. In addition to various PET/CT models available, the acquisition and reconstruction parameters applied at different sites vary greatly due to local preferences [24, 32, 33]. Centres equipped with PET systems having new acquisition and reconstruction technologies available, often tend to aim for the possible best lesion detection, which may not be in line with quantitative harmonising standards [34]. The aforementioned technical factors impose a significant source of variability in PET quantification [24, 32] that should be addressed by the international community.

Numerous professional societies and organisations such as the Society of Nuclear Medicine and Molecular Imaging (SNMMI), American College of Radiology Imaging Network (ACRIN), Radiological Society of North America - Quantitative Imaging Biomarkers Alliance (RSNA-QIBA), The American Association of Physicists in Medicine (AAPM) and the European Association of Nuclear Medicine (EANM) are promoting harmonisation of imaging procedures [35–37] in order to reduce the variability of PET image quantification in a multicentre setting. Many of these programs rely on quality control procedures utilising standard phantoms [38] for standardisation of quantification [32, 39–41] and harmonisation of PET/CT systems [35]. Review papers on describing some of the results and experience in running such programs have been published by Scheuermann et al. [39] and more recently by Sunderland et al. [32].

In 2006, the European Association of Nuclear Medicine (EANM) launched the EANM Research Ltd. (EARL) initiative. One of the main objectives of the program has been promoting multicentre nuclear medicine and research. In 2010, the FDG-PET/CT accreditation program was created in order to address variability in the quickly growing field of quantitative FDG-PET imaging by setting up guidelines and specifications to which the participating sites must adhere. The bandwidths for the current EARL specifications were developed during a pilot study in 2010–2011 involving 12 PET/CT systems. Based on this study, specifications for SUVmean and SUVmax recovery coefficients were derived, which accommodated all investigated systems. From its initiation until July 2016, EARL has collected approximately 2500 phantom datasets from more than 200 PET/CT systems from over 150 imaging sites worldwide. The data analysed by EARL encompasses the majority of the system types in clinical use over the past 10 years and incorporates sites with various backgrounds giving it a broad basis to represent the field as a whole.

The objective of this paper is to report the findings obtained so far in the EARL standardisation program and their impact on the quantitative variability of accredited PET/CT systems. Analysis of phantom scans from the largest number of active PET centres so far provides representative details of current quantitative capabilities of FDG-PET imaging and the variability to be expected. Understanding the characteristics of variability and the impact on standardisation will help planning multi-centre clinical trials, utilising quantitative FDG-PET/CT imaging and advance use of PET as a quantitative imaging biomarker.

The secondary objective of this study is to explore ways to improve the EARL FDG-PET accreditation program based on the retrospective analysis of phantom data collected in the EARL database.

## Materials and methods

### Acquisition and submission of data to EARL

Sites, which are seeking EARL FDG-PET/CT accreditation for the first time, need to pass the initial procedure. This procedure includes the submission of an online questionnaire and a signed statement – these documents have to be submitted at the start of the accreditation procedure and revised annually, whereas QC documents need to be regularly provided in order to maintain the EARL accredited status.

For the first and follow-up procedures, sites have to perform calibration QC and image quality QC measurements. The calibration QC measurements have to be repeated every 3 months and image quality QC procedures annually, while the data needs to be provided to EARL upon completion of the procedures. During each round of QC survey, there is a 3 week period for the sites to collect the data and submit it to EARL, followed by a 3 week period of analysing the data by EARL and reporting the results back to the sites.

For the calibration QC measurements, centres are asked to use a cylindrical phantom with the following characteristics: diameter of about 20 cm (17 to 22 cm) and length sufficient to cover the entire axial field of view (FOV). Furthermore, the exact volume of the calibration phantom should be known and recorded in the calibration QC scan report form. The phantom has to be filled with water and about 70 MBq ^18^F–FDG added to it, aimed at expected phantom acquisition time.

For image quality QC measurements, the NEMA NU2–2007 image quality phantom is required. The phantom has a fillable torso cavity to act as a background compartment, a 5 cm diameter cylindrical lung insert in the centre and six fillable spheres with internal diameters of 10 mm, 13 mm, 17 mm, 22 mm, 28 mm and 37 mm positioned coaxially around the lung insert. The phantom background compartment and the spherical inserts have to be filled with ^18^F–FDG solution aimed at activity concentrations at the start of the PET scan of 2 kBq/mL and 20 kBq/mL, respectively, resulting in a sphere to background ratio of 10:1.

With both phantoms, routine quantitative whole body PET/CT scans have to be performed with two PET bed positions of at least 5 min each, including a (low dose) CT for attenuation correction purposes [35]. After reconstruction, the attenuation corrected PET, non-attenuation corrected PET and CT images of the phantoms have to be uploaded into the EARL central database, along with scan report forms.

### Quantitative analysis and approval by EARL

The uniform calibration QC phantom and NEMA NU2 IQ body phantom images uploaded into the EARL database are evaluated centrally, making use of a standardised semi-automatic quantitative analysis tool developed internally within EARL. The software uses activity and time information provided by the scan report forms. The average volumetric SUV bias is generated as relative deviation between measured and calculated activity concentration values (Eq. 1). The SUV recovery coefficients (RCs) for the six spherical inserts are based on 50% background corrected isocontour VOI (RC_SUVmean_) and maximum voxel value included in the VOI (RC_SUVmax_).

1 $$ SUVbias\ \left(\%\right)=\left(\frac{C_{measured}}{C_{calculated}}-1\right)\times 100\%; $$,

where

$$ {C}_{measured}- activity concentration measured from images $$

$$ {C}_{calculated}- activity concentration calculated from injection data $$

EARL is applying SUV bias and RC values acceptance criteria, which were defined by feasibility studies performed on the systems used in clinical practices at the start of the standardisation - a study is underway in order to update these. When approval is not granted, the site undergoing (re-)accreditation is asked to take corrective actions, for example: recalibration of the PET system, adjustment of reconstruction parameters, repeating the phantom scan and so on. When required, EARL is advising the sites. A Manual describing the accreditation program in detail as well as information on the EARL website [42] is also available. If submitted QC documents meet the standard requirements, FDG-PET/CT accreditation is granted, and the department is listed on the EARL website (http://earl.eanm.org) as an accredited PET/CT centre of excellence. Furthermore, the site is provided with an accreditation certificate and signet, which can be used on its correspondence and website.

### Data clean-up and preparation

To allow for data extraction, the EARL database had to be cleaned of duplicates and entries with insufficient or missing information removed, entry errors were identified and the individual site identification data ignored thereby providing an anonymised set of data for evaluation. First and subsequent site submissions were identified and marked as such.

### Analysis

The calibration QC and image quality QC datasets from the EARL database will be analysed based on the type of the phantom, accreditation approval status, manufacturer of the PET/CT system and whether it was the first or a subsequent QC data submission. The SUV bias and normalised SUV biases were analysed as well as the recovery coefficients for each sphere size, separately for SUVmean and SUVmax. For each parameter, mean, median, standard deviation, standard error and skewness were calculated. Longitudinal reproducibility analysis was performed on 16 systems (systems A to P) selected based on each having sufficient longitudinal data of at least 18 approved CalQC data submissions or at least five approved IQQC datasets.

## Results

### General overview

**Fig. 1:**
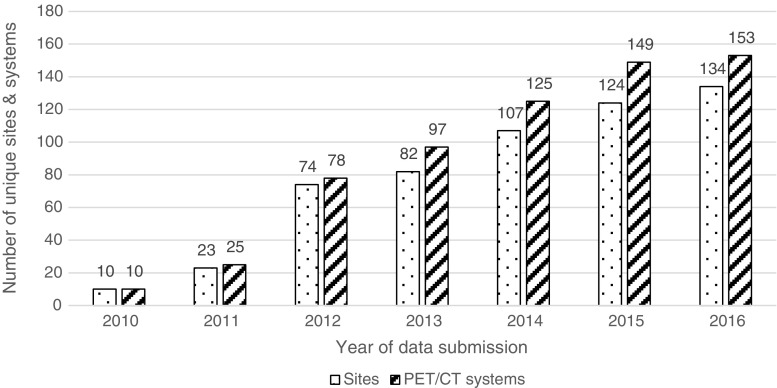
Number of sites and PET/CT systems participating in the EARL accreditation program. For 2016 data has been collected from January to July

Data reviewed in this paper encompasses all submissions to the EARL database from the initiation of the standardisation program in November 2010 to July 2016. Figure 1 represents the number of sites and systems participating each year. After correcting for erroneous, partial and duplicate entries, 1816 CalQC and 778 IQQC datasets were used for further analysis. The datasets were 29% (*n* = 752) from GE-, 29% (*n* = 741) from Philips- and 42% (*n* = 1101) from Siemens-systems.

First data submissions constitute 10% (*n* = 175) of all CalQC and 23% (*n* = 178) of all IQQC scans. 85% (*n* = 149) of the first and 94% (*n* = 1537) of subsequent CalQC data submissions could be approved by EARL. This results in an overall approval rate for CalQC of 93% (*n* = 1686). Table 1 states descriptive statistics for CalQC initial and subsequent submissions.

**Table 1:** CalQC SUV bias statistics from first, regular ongoing and all EARL approved submissions (pooled and per vendor)

CalQC	Mean SUV bias (%)	Median SUV bias (%)	SUV bias Std. Dev (%)	Skewness	Submissions with SUV bias below EARL specs	Submissions with SUV bias above EARL specs	Submissions with SUV bias within EARL specs
All CalQC	−1.14 (±0.13)	−1.01	5.36	−0.32	3%	2%	95%
All approved CalQC	−0.97 (±0.09)	−0.94	3.71	0.15	0%	0%	100%
Sites’ first submitted CalQC	−1.25 (±0.46)	−0.79	6.06	−1.14	6%	3%	91%
Subsequent approved submissions CalQC	−1.01 (±0.09)	−1.02	3.66	0.16	0%	0%	100%
All approved GE CalQC	−1.53 (±0.15)	−1.60	3.27	0.31	0%	0%	100%
All approved Philips CalQC	−1.78 (±0.18)	−1.71	3.89	0.26	0%	0%	100%
All approved Siemens CalQC	−0.05 (±0.14)	0.06	3.65	0.01	0%	0%	100%

Out of all systems (*n* = 200) that have enrolled in the program, the accreditation for 47 systems (24%) has been discontinued for various reasons, such as scanner replacement or stopped participation in trials requiring EARL accreditation.

### Calibration QC

**Fig. 2:**
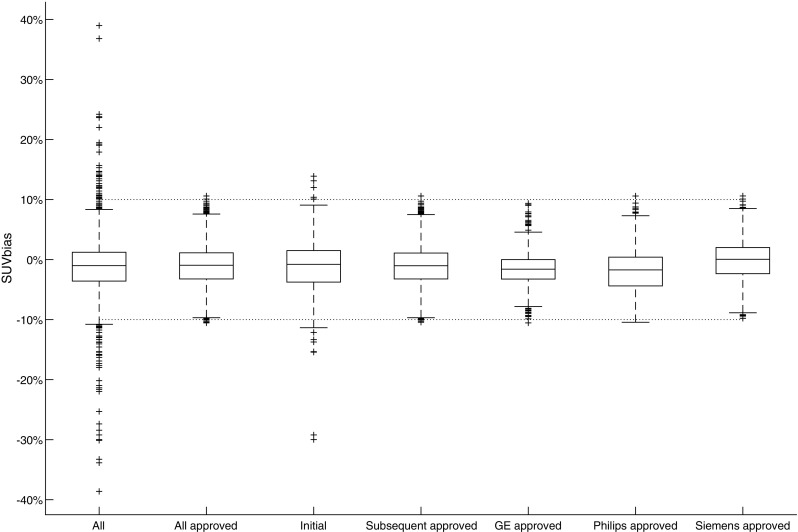
Comparison of CalQC SUV bias distribution for all, initial and subsequent submissions along with vendor based distribution of approved results. The dotted horizontal lines represent EARL specification limits. Central line of the box is the median, edges of the box are the 25th and 75th percentiles, the whiskers extend to either of the most extreme data points, which are not considered outliers or 1.5 times interquartile range. The outliers are marked using plus signs

**Fig. 3:**
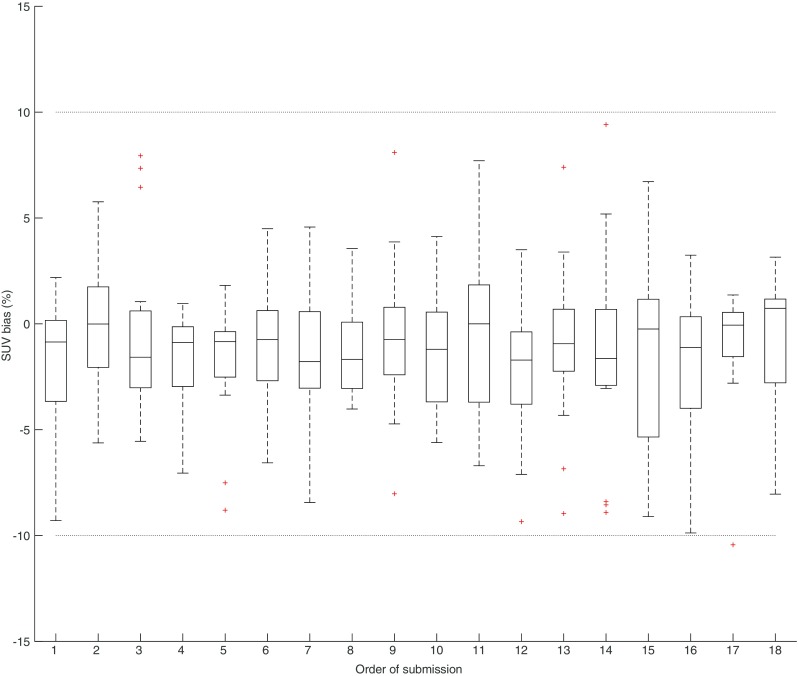
Longitudinal plots of EARL approved CalQC results from the 16 systems. SUV bias deviation from the expected value. The dotted horizontal lines represent EARL specification limits. Central line of the box is the median, edges of the box are the 25th and 75th percentiles, the whiskers extend to either of the most extreme data points, which are not considered outliers or 1.5 times interquartile range. The outliers are marked using plus signs

Detailed descriptive statistics for CalQC SUV bias are summarised in Table 1. Figure 2 demonstrates CalQC SUV bias distribution for all, initial and subsequent submissions along with vendor based distribution of approved results. It was found 3% (*n* = 60) of all CalQC submissions were below and 2% (*n* = 36) above the corresponding EARL SUV bias limits of −10% and +10%. Also, 9% (*n* = 16) of systems could not be approved at first CalQC submission, but after corrective actions all of the scanners fulfilled the EARL specifications. Significant mean SUV biases of −1.53% (*p* < 0.0001) and −1.78% (p < 0.0001) were observed in approved datasets from GE and Philips systems, respectively, while datasets from Siemens systems did not demonstrate this deviation. In Fig. 3 longitudinal CalQC volumetric SUV bias is plotted as a function of the order of subsequent submissions.

### Image quality QC

**Fig. 4:**
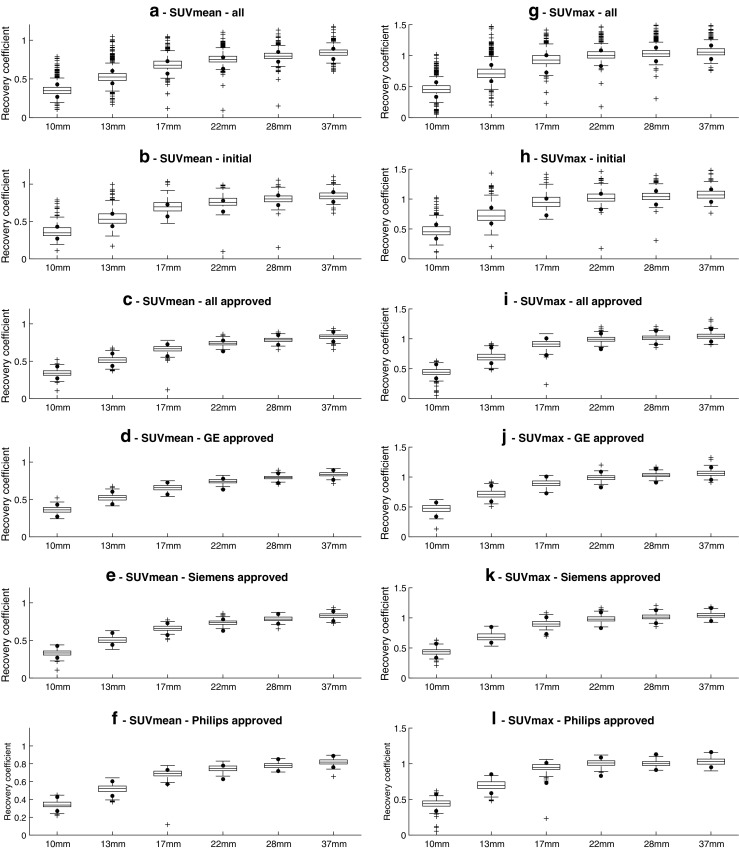
IQQC SUVmean (**a** to **f**) and SUVmax (**g** to **l**) recovery results, regular ongoing and all EARL approved submissions (pooled and per vendor). Dots represent EARL specification limits. Central line of the box is the median, edges of the box are the 25th and 75th percentiles, the whiskers extend to either of the most extreme data points which are not considered outliers or 1.5 times interquartile range. The outliers are marked using plus signs

Descriptive statistics for IQQC SUVmax and IQQC SUVmean results for each sphere size are summarised in Supplemental Tables 1 and 2. Figure 4 shows the distribution of sphere recoveries of all submitted data and approved IQQC submissions along with SUV recovery distributions separately per manufacturer. A large variability in sphere recoveries was observed. Out of all sphere recoveries from IQQC datasets, 30% (*n* = 1381) of SUVmean and 23% (*n* = 1095) of SUVmax recoveries failed to meet the EARL accreditation criteria. After corrective actions the corresponding values dropped to 12% (*n* = 360) and 9% (*n* = 254) respectively. A positive bias in the recovery values was observed prior to and also after the corrective actions and granted accreditation, respectively. 47% (*n* = 84) of sites’ initial IQQC submissions and 68% (*n* = 409) of all consecutive IQQC submissions were approved by EARL. Overall approval rate for IQQC submissions was 63% (*n* = 493).

**Fig. 5:**
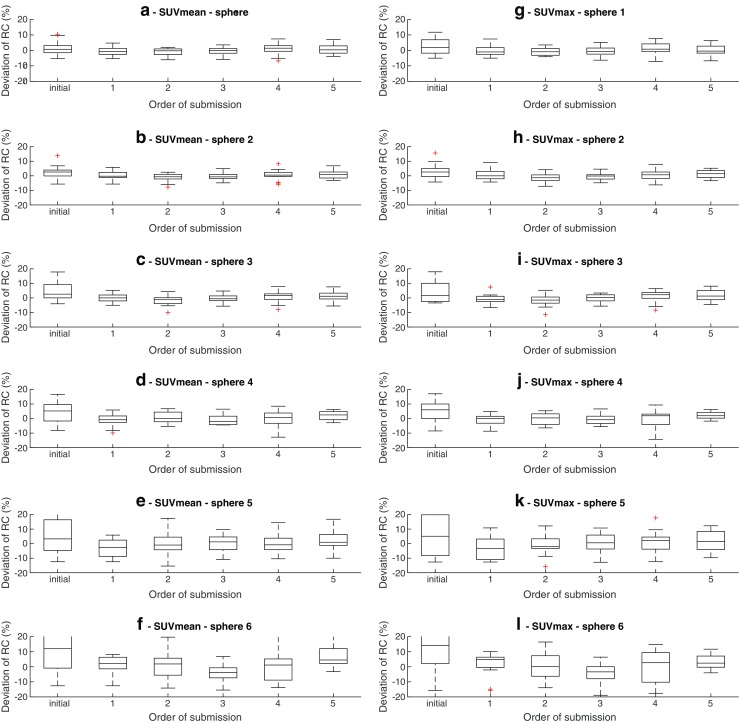
Longitudinal analysis of IQQC results from 16 scanners. Recovery coefficients biases from the mean of the respective scanner. "Initial“data series represents the first IQQC submission for each system. Central line of the box is the median, edges of the box are the 25th and 75th percentiles, the whiskers extend to either of the most extreme data points which are not considered outliers or 1.5 times interquartile range. The outliers are marked using plus signs

To evaluate the longitudinal performance of the 16 systems, sphere recoveries for all sphere sizes were plotted based on the order of subsequent submissions, as seen in Fig. 5. From the figure, it can be seen that for each sphere there is an initial large variability in observed SUV recoveries, which is reduced and becomes harmonised during subsequent submissions.

## Discussion

The number of sites participating in the EARL accreditation program is steadily increasing and the received data is almost equally distributed among the three major PET/CT system manufacturers.

Inconsistency was observed in the names provided for same types of systems and in some cases the device serial numbers were missing or had been changed at the occasion of software upgrade or service maintenance. This complicated the distinction between new systems and those already existing in the EARL database. As a solution, the EARL database client portal could be configured so that the system type selection be performed by choosing from a predefined list, in the same way as it currently works for system manufacturer, permitting that the regularly submitted QC data be checked and, if necessary, corrected for constancy of the core data and the device serial number in particular.

A small number (*n* = 19) of calibration QC submissions were not included in further analysis due to large SUV biases observed. The high values might have been related to improper data communication from the participants, inhomogeneous phantoms or system failures left unnoticed by the centres. In all these cases, sites were asked to implement corrective actions and redo the experiments. As a result, all of the affected systems achieved compliance with the EARL specifications and were granted accreditation.

Regardless of manufacturer or model, all systems were able to comply with the CalQC specifications set up by EARL. Only 9% of sites first QC submissions and 5% of all CalQC submissions demonstrated a measured activity bias of more than 10%. Scheuermann et al. in their review of ACRIN PET Core Laboratory program reported a similar initial SUV or normalisation calibration failure rate of 12% within the same acceptance criteria of SUV 1.0 ± 0.1 [39].

Compared to all received data, the non-compliances were almost eliminated in EARL approved data. In the datasets reviewed and approved by EARL, all CalQC SUV biases fall within the range of ±10.5%, which aligns with the target of ±10%. Although the fraction of non-approved data from combined first and regular submissions was relatively small (about 5%), the importance of this fact should not be underestimated, since noncompliance in calibration QC procedure means gross error in basic system calibration, which would affect all further PET quantitative evaluations.

CalQC average values demonstrate a slight but statistically significant underestimation of the activity concentration or SUV by Philips and GE systems, while data from Siemens devices do not show this deviation. Scheuermann et al. reported similar results for Philips systems [39]. Whether this is due to some systematic differences among the vendors’ calibration procedures, drifts in calibration values or some unknown effects, could not be derived from the current data but should be subject for further investigations. However, it is important to note that these errors were well within 2% and are very likely to be clinically irrelevant.

**Fig. 6:**
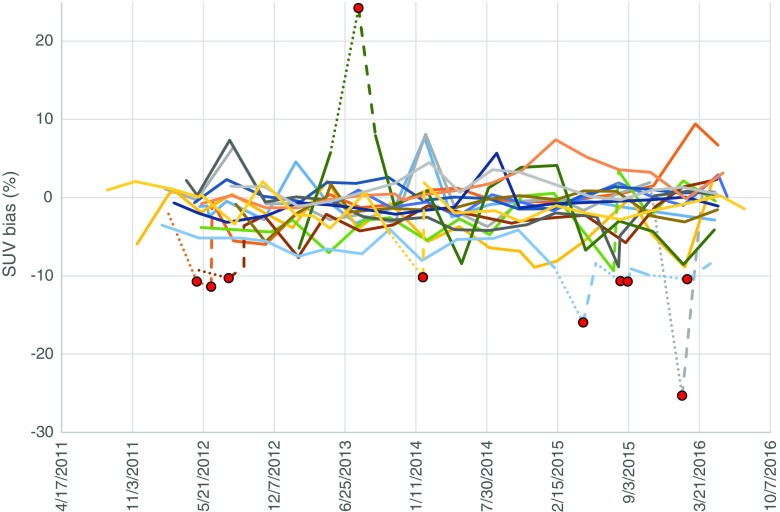
Longitudinal analysis of the 16 systems’ CalQC results. SUV bias values for each system are presented as separate lines. Dotted lines represent outliers and dashed lines subsequent corrective actions. Red dots represent data points outside EARL specifications

Longitudinal analysis of the CalQC results from 16 selected systems, visualised in Fig. 6, illustrates good performance of the systems and reflects the quantification stability achieved by the accredited sites. Large SUV biases were only occasionally observed, and were all corrected by the sites after receiving notification from EARL. In most of the cases, corrective action had been taken within 4 weeks.

The majority of PET/CT systems followed throughout the investigation period showed good reproducibility of the CalQC results seen in Fig. 3. Longitudinal SUV bias values lie mostly within ±5% of the expected value, which is in agreement with data published by Geworski et al. [43] and more recently by Sunderland et al. [32]. The rest of the systems also meet the ±10% calibration accuracy criteria. Our findings suggest opportunities for the implementation of stricter accreditation specification for EARL CalQC SUV biases.

The comparison between all received and EARL approved data indicates significant reduction of outliers for IQQC results (Supplemental Tables 1 and 2). However, some of the data not strictly within EARL specifications was still accepted after critical review. This means the noncompliant data was deemed acceptable in case only one of the spheres being slightly out of the specifications, but the overall shape and magnitude of RC curve could be determined to a sufficient extent and being compliant with EARL criteria. Moreover, in these cases submissions were reviewed retrospectively and consistency of acquisition, reconstruction and settings was verified. While in case of the larger diameter spheres meeting EARL specifications was common, for the smallest sphere (10 mm diameter) SUVmax data remained outside of specified bandwidth in 26% of the EARL approved submissions. Achieving harmonised quantitative results for the smallest sphere turned out to be challenging due to its increased sensitivity to image noise and phantom positioning within the PET field of view.

Although in most cases when the spheres’ RCs did not meet the specifications, sites were asked to redo experiments, still a noticeable upward bias in results for the smallest sphere could be observed. Therefore, based on these findings EARL implemented slightly adjusted recovery specifications for the smallest sphere starting from the 1st of January 2017.

Prior to harmonisation, the average recoveries of all spheres demonstrated a positive bias compared to EARL specifications. The trend could still be observed to some extent within the approved results. This can be attributed to both the vendors and sites preferences leaning towards reconstruction algorithms emphasising contrast and detectability. The positive bias in the case of the 10 mm diameter sphere might also be attributed to newly adopted acquisition and reconstruction technologies (e.g., including resolution modelling), which aims at increasing small lesion detectability. This trend was also observed by Sunderland et al. who showed that recoveries were generally higher for systems employing TOF and PSF reconstructions [32]. Since more modern PET/CT systems with new technologies appear in the field, a review of the existing EARL specifications is required in order to facilitate the inclusion of the increased contrast recovery capabilities of these systems. EARL is currently carrying out a feasibility study aiming at redefining the accreditation criteria by taking into account not only the new technologies but also considering that the majority of the PET/CT systems currently in clinical use should be able to comply.

By comparing the first and following regular submissions from participating sites, a relative increase can be observed in meeting the EARL specifications which is expected since the accredited sites gain experience in performing quantitative calibrations and assessment of their systems. The staff’s increased awareness towards the necessity of regular calibration and quality control of the systems is one of the benefits of participation in an accreditation/standardisation program, which is difficult to measure but should not be underestimated.

IQQC longitudinal analysis results, presented in Fig. 5 visualise the major improvement of regular submissions in relation to the first submission. As the sphere size decreases, the improvement becomes more prominent. After initial adjustment of the reconstruction parameters, the longitudinal reproducibility remains stable and generally within ±10% for the largest four spheres and ±20% for the smallest two spheres. These findings emphasise that PET/CT performance and the execution of the QC experiments show high reproducibility and demonstrate that long-term maintenance of a harmonised PET/CT network is feasible and achievable.

## Conclusion

The European Association of Nuclear Medicine (EANM) has been running an FDG-PET/CT accreditation program under the EANM Research Ltd. (EARL) initiative in order to harmonise quantitative PET/CT performance and facilitate multi-centre nuclear medicine and research. The number of sites and systems participating in the accreditation program has steadily increased over the years with similar numbers of scanners from each of the three major vendors.

Outliers observed in the overall submissions of both CalQC and IQQC were largely eliminated in subsequent submissions after notification from EARL. Excellent longitudinal performance was observed in most of the systems – a majority demonstrated CalQC values reproducible within 5% and IQQC results within 10% for the largest four spheres and 20% for the smallest two spheres. Occasional deviations from expected values were rapidly resolved by the sites after notification from EARL. Regardless of system manufacturer or model, all vendors were able to comply with the accreditation specifications set out by EARL.

Prior to harmonisation, IQQC data demonstrated a slight positive bias relative to EARL specifications, which suggested carrying out a review and an update in order to account for the advances in acquisition and reconstruction technologies in PET/CT.

In this manuscript we have demonstrated that the EARL accreditation program can successfully identify gross PET/CT calibration errors and reduces variability in PET/CT performance by longitudinally performing harmonisation QC experiments. The program is running successfully for more than 6 years and shows that a harmonising accreditation procedure is feasible and achievable.

Centres with accredited PET/CT systems benefit greatly from participating in large-scale accreditation programs, which facilitate the implementation of procedural guidelines widely recognised by the imaging community.

## Electronic supplementary material

ESM 1 (DOCX 20.0 kb)
