# Supplementary material for: EANM/EARL FDG-PET/CT accreditation - summary results from the first 200 accredited imaging systems
Source: Eur J Nucl Med Mol Imaging. 2017 Dec 1;45(3):412–22. doi: 10.1007/s00259-017-3853-7 (PMC5787222; doi:10.1007/s00259-017-3853-7)
Supplement: Supplementary file 1 — (DOCX 20.0 kb) [file 259_2017_3853_MOESM1_ESM.docx]

# Supplement

| **IQQC** | **Mean recovery coefficient** | **Median** | **Standard Deviation** | **Skewness** | **% of submissions with RC below Earl specs** | **% of submissions with RC above Earl specs** | **% of submissions with RC within Earl specs** |
| --- | --- | --- | --- | --- | --- | --- | --- |
| All 37mm sphere SUVmax | 1.08 (±0.01) | 1.06 | 0.156 | 12.9 | 6% | 13% | 81% |
| Approved 37mm sphere SUVmax | 1.05 (±0.01) | 1.04 | 0.160 | 18.5 | 2% | 2% | 96% |
| All 28mm sphere SUVmax | 1.05 (±0.01) | 1.03 | 0.150 | 12.7 | 4% | 13% | 83% |
| Approved 28mm sphere SUVmax | 1.03 (±0.01) | 1.02 | 0.154 | 18.9 | 0% | 2% | 98% |
| All 22mm sphere SUVmax | 1.02 (±0.01) | 1.01 | 0.144 | 10.2 | 1% | 17% | 83% |
| Approved 22mm sphere SUVmax | 1.00 (±0.01) | 0.99 | 0.140 | 17.9 | 0% | 3% | 97% |
| All 17mm sphere SUVmax | 0.95 (±0.01) | 0.93 | 0.152 | 6.9 | 2% | 23% | 75% |
| Approved 17mm sphere SUVmax | 0.92 (±0.01) | 0.91 | 0.138 | 14.1 | 1% | 8% | 92% |
| All 13mm sphere SUVmax | 0.73 (±0.01) | 0.71 | 0.168 | 3.6 | 11% | 14% | 75% |
| Approved 13mm sphere SUVmax | 0.70 (±0.01) | 0.69 | 0.123 | 11.9 | 6% | 2% | 92% |
| All 10mm sphere SUVmax | 0.47 (±0.01) | 0.45 | 0.139 | 3.2 | 6% | 32% | 62% |
| Approved 10mm sphere SUVmax | 0.45 (±0.01) | 0.44 | 0.108 | 8.8 | 3% | 23% | 74% |

**Supplemental table 1.** IQQC SUVmax statistics for all sphere sizes calculated for all received and EARL approved submissions

| **IQQC** | **Mean recovery coefficient** | **Median** | **Standard Deviation** | **Skewness** | **% of submissions below Earl specs** | **% of submissions above Earl specs** | **% of submissions within Earl specs** |
| --- | --- | --- | --- | --- | --- | --- | --- |
| All 37mm sphere SUVmean | 0.85 (±0.01) | 0.84 | 0.112 | 15.0 | 7% | 18% | 76% |
| Approved 37mm sphere SUVmean | 0.83 (±0.01) | 0.83 | 0.120 | 19.4 | 2% | 4% | 94% |
| All 28mm sphere SUVmean | 0.80 (±0.01) | 0.79 | 0.111 | 13.9 | 7% | 17% | 76% |
| Approved 28mm sphere SUVmean | 0.79 (±0.01) | 0.79 | 0.116 | 19.6 | 2% | 3% | 95% |
| All 22mm sphere SUVmean | 0.76 (±0.01) | 0.75 | 0.107 | 11.3 | 1% | 32% | 67% |
| Approved 22mm sphere SUVmean | 0.75 (±0.01) | 0.74 | 0.106 | 18.7 | 0% | 14% | 86% |
| All 17mm sphere SUVmean | 0.69 (±0.01) | 0.68 | 0.111 | 7.0 | 4% | 25% | 71% |
| Approved 17mm sphere SUVmean | 0.67 (±0.01) | 0.67 | 0.101 | 14.4 | 1% | 9% | 89% |
| All 13mm sphere SUVmean | 0.54 (±0.01) | 0.52 | 0.118 | 4.0 | 10% | 17% | 73% |
| Approved 13mm sphere SUVmean | 0.52 (±0.01) | 0.52 | 0.088 | 12.9 | 5% | 4% | 90% |
| All 10mm sphere SUVmean | 0.36 (±0.01) | 0.35 | 0.105 | 2.5 | 10% | 30% | 60% |
| Approved 10mm sphere SUVmean | 0.34 (±0.01) | 0.34 | 0.082 | 7.2 | 7% | 21% | 73% |

**Supplemental table 2.** IQQC SUVmean statistics for all sphere sizes calculated for all received and EARL approved submissions
